# Supplementary material for: Dripplons as localized and superfast ripples of water confined between graphene sheets
Source: Nat Commun. 2018 Apr 16;9:1496. doi: 10.1038/s41467-018-03829-1 (PMC5902618; doi:10.1038/s41467-018-03829-1)
Supplement: Supplementary file 1 — Supplementary Information [file 41467_2018_3829_MOESM1_ESM.pdf]

Supplementary Information

Dripplons as localized and superfast ripples of water confined between graphene sheets

Yoshida *et al.*

Supplementary Information of "Dripplons as localized and superfast ripples of water confined between graphene sheets"

Hiroaki Yoshida[1,2], Vojtěch Kaiser[1], Benjamin Rotenberg[3], Lydéric Bocquet[1]

[1]Laboratoire de Physique Statistique, UMR CNRS 8550, Ecole Normale Supérieure, PSL Research University, 24 rue Lhomond, 75005 Paris, France

[2]Toyota Central R&D Labs., Inc., Nagakute, Aichi 480-1192, Japan

[3]Sorbonne Université, CNRS, Physicochimie des électrolytes et nanosystèmes interfaciaux, UMR PHENIX, F-75005, Paris, France

Supplementary Note 1: Molecular dynamics simulations

In our molecular dynamics (MD) simulations, we consider a system with N water molecules confined between two graphene sheets. The graphene sheets span the entire simulation box in the lateral directions, x and y, and we use the periodic boundary conditions in these directions. The TIP4P/2005 potential[1] is employed to model water molecules and the Dreiding force field[2] is used for graphene sheets. The Lennard-Jones (LJ) parameters for cross interactions are determined by means of the Lorentz–Berthelot mixing rules.[3]

All the MD simulations are implemented using the open-source code LAMMPS.[4] The time integration is carried out with a time step of 1 fs and the temperature is maintained at 300 K using the Nosé–Hoover thermostat with a time constant of 0.2 ps. We have checked that the temperature of the water between the graphene sheets is properly controlled by the thermostat applied in the reservoirs. The water molecules are maintained rigid using the SHAKE algorithm.[5] The LJ interactions are computed using a spherical cut-off 9.8 Å, while long-range Coulomb interactions are treated with the particle-particle particle-mesh (PPPM) method and applying a slab correction[6] to deal with the non-periodicity in the z direction.

The graphene sheets are assumed to be rigid planes in obtaining the data shown in Fig. 2. The constant normal pressure condition in Fig. 2**a** is realized by tuning the force acting on the graphene sheets directly. More precisely, while one of the graphene sheets is completely frozen, the atoms belonging to the other sheet are constrained such that they move only in the z direction as a rigid body, and the common force acting on each atom is determined so that the force per unit surface corresponds to the applied pressure. The data shown in Figs 2**b** and **c** are obtained from the simulations with both graphene sheets being fixed at the specified inter-layer distance h. The pressure on the graphene is obtained by measuring the force acting on all the atoms in the graphene sheets.

The size of the graphene sheets for the main data shown in Fig. 2**a** is $S = 3.9 \times 4.3\,\mathrm{nm}^2$. As discussed in the main text, the smaller size constrains the system to remain homogeneous, *i.e.* it prevents the coexistence between water mono- and bilayers (red-dashed line in Fig. 2**a**). To check the effect of larger sizes, we carried out the same simulations for different graphene sheets. Supplementary Figure 1**a** shows the data obtained for the sizes $S = 7.9 \times 8.5\,\mathrm{nm}^2$ and $15.7 \times 17.0\,\mathrm{nm}^2$, on top of the same data shown in Fig. 2**a**, which clearly shows essentially identical results.

The data shown in Figs. 1, 3-5 are obtained with the rigidity of graphene sheets being released. As mentioned above, we use the Dreiding force field[2] for the interaction potential between carbon

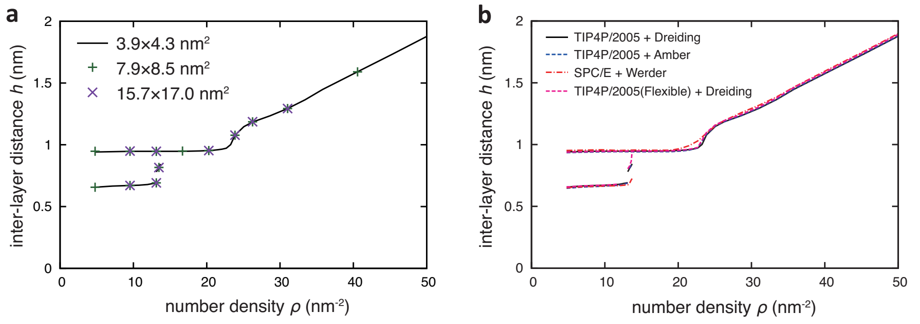

Supplementary Figure 1: **a**: Inter-layer distance h versus number density ρ, obtained for the system of water molecules between two rigid graphene sheets of $S = 3.9 \times 4.3\,\mathrm{nm}^2$, $7.9 \times 8.5\,\mathrm{nm}^2$, and $15.7 \times 17.0\,\mathrm{nm}^2$, under a pressure of 1 atm. **b**: The same diagram for the graphene sheets of size $S = 3.9 \times 4.3\,\mathrm{nm}^2$, obtained using the set of TIP4P/2005 water molecules and Dreiding force field, the set of TIP4P/2005 water molecules and Amber force field, the set of SPC/E and the Werder potential, and the set of TIP4P/2005-Flexible water molecules and Dreiding force field.

atoms. The bonds, angles, and dihedrals are defined within each flexible graphene sheets; note that these are also defined across the periodic boundaries. There is no lateral tension when the graphene is perfectly planar. The properties of the Dreiding potential have recently been carefully validated in Supp. Ref. 7. On top of them, the bending rigidity of a single graphene sheet is checked in the present study, because it plays a critical role in the creation of the dripplon. We measured the bending energy using stationary molecular simulations. Specifically, we consider a graphene sheet with a finite width; one end is fixed and a bending moment M (per unit length) is applied to the other end of the sheet. From the measured curvature κ of the sheet, we compute the bending rigidity as $B = M/\kappa$. We obtained $B = 2.2\,\mathrm{eV}$ for the bending along the zigzag direction, and $B = 2.1\,\mathrm{eV}$ along the armchair direction. The bending rigidity was also evaluated by means of the MD for vibrating sheets in Supp. Ref. 8, where the same value $B = 2.1\,\mathrm{eV}$ was reported. Although the direct experimental measurement of bending stiffness of mono-layer graphene has not been reported in the literature, these values are in good agreement with the often-cited experimental value $1.2\,\mathrm{eV}$.[9]

In the case of flexible graphene, the pressure acting on the sheets is controlled by the water reservoirs located above and below the graphene sheets, as shown in Fig. 1. Rigid graphene sheets are placed at each end of the reservoirs. They are used as pistons to control the pressure in the reservoirs. The homogeneous initial condition, as shown in Fig. 3**a**, is prepared by running a simulation while freezing the graphene sheets of small size (typically $S = 1.2 \times 1.3\,\mathrm{nm}^2$, to avoid the bubble formation shown in Fig. 2**a**) and replicating the equilibrium state to the desired size. The size of the sheets in Fig. 3**a** is $S = 22.1 \times 23.0\,\mathrm{nm}^2$. The height of each reservoir is initially 2 nm. The number of atoms in one flexible graphene sheet is 18468, and that of the confined water molecules is 6156. The total number of water molecules including the reservoirs is 52812.

We checked the effect of the interaction potential of water molecules and carbon atoms. As described above, in the present paper we use the TIP4P/2005 force field for water molecules[1] with the Dreiding force field[2] for the carbon atoms, which has carefully been validated in Supp. Ref. 7. In addition, for the rigid graphene case, we examined the diagram in Fig. 2**a** for the combinations of TIP4P/2005 water molecule with Amber force field,[10] SPC/E water molecule[11] with the Werder

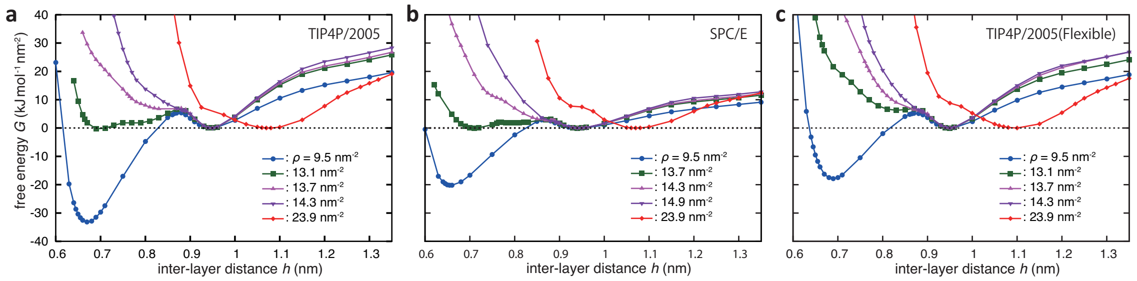

Supplementary Figure 2: Free energy per unit area G at several values of ρ; **a**: TIP4P/2005 water model with Amber potential for water-carbon interaction (Fig. 2b of the paper), **b**: SPC/E water model with Werder potential, and **c**: TIP4P/2005(Flexible) water model with Dreiding potential. The reference energy for each value of ρ is chosen such that $G = 0$ at the local minimum in $h > 0.9\,\mathrm{nm}$.

Supplementary Table 1: Size of dripplons at $\rho_{\mathrm{av}} = 12.53\,\mathrm{nm}^{-2}$ in $S = 15.7 \times 17.0\,\mathrm{nm}^2$ for different sets of interaction potentials.

TIP4P/2005 - Dreiding	3.3 nm
TIP4P/2005 - Amber	3.3 nm
SPC/E - Werder	2.4 nm

potential,[12] and TIP4P/2005-Flexible water molecule[13] with Dreiding force field, which are also widely used in the literature. The results are compared in Supp. Fig. 1**b**. The main behavior of the layered structure of the confined water molecules is unchanged, including the coexistence of the water mono-layer (with vacancy) and the bi-layer. We carried out further computations to calculate the free energy G to show that the single- and double-layer structures are stable for different water models, *i.e.* SPC/E and TIP4P/2005-Flexible models on top of the TIP4P/2005. The results are shown in Supp. Fig. 2. Though there are indeed slight quantitative difference, the important features, *i.e.* the thermodynamic stability of single and double layer structures are clearly observed in all cases. The creation of the dripplon is also examined for these sets of potential. We performed simulations for the setup of the flexible graphene sheets; the flexibility of graphenes is still described by the Dreiding force field. The obtained radius of the dripplon is identical with to that obtained with TIP4P/2005 and Amber, but there is a quantitative difference with the set of SPC/E and Werder potential. Since the cohesion of water molecules plays an essential role in the creation of a dripplon, the choice of model for water molecules is expected to result in (slight) quantitative differences. Nevertheless, the fact that the dripplon is indeed created for all these sets confirms the robustness of the phenomenon.

Supplementary Note 2: Analysis of the spinodal decomposition

The theory of the stability of thin liquid films follows Supp. Refs. 14, 15. As discussed in the main text, the stationary homogeneous film considered here is hypothetical because the forces driving the corrugation growth are unbalanced, and therefore the film is unstable. The theory

predicts the wavelength at which the longitudinal fluctuation grows fastest as:

$$\Lambda_m = 2\pi\xi, \tag{1}$$

$$\xi = \left(\frac{\sigma}{\partial\Pi/\partial h}\right)^{1/2}, \tag{2}$$

with the typical time scale

$$\tau_m = \frac{24\sigma\eta}{h^3}\left(\partial\Pi/\partial h\right)^{-2}, \tag{3}$$

where σ is the surface tension of the liquid film, Π is the disjoining pressure, h is the film thickness, and η is the viscosity of the liquid.

We applied this theory to our system and estimated the wavelength corresponding to Supp. Eq. (1) as $\Lambda_m = 5.7\,\mathrm{nm}$, using the snapshots at $t = 5\,\mathrm{ps}$. The decomposition process observed around $\sim$ps is consistent with the time scale of the order of 10 ps predicted by Supp. Eq. (3). Here, we detail the procedure for obtaining the scattering intensity function shown in Fig. 3**b**, to characterize the growth wavelength of the fluctuation in the spinodal decomposition process. This procedure follows that used in Supp. Ref. 16 to characterize porous structures. We first visualize the dripplons in terms of the gap distribution as shown in Supp. Fig. 3**a** (also in Fig. 3**a**). Then the binary images as in Supp. Fig. 3**b** are generated with the threshold at 9.0 Å, *i.e.* the dripplon is defined as the region in which $h > 9.0$ Å. Let $Z(\boldsymbol{x})$ be a characteristic function defined for the binary images, taking the value of unity if $\boldsymbol{x}$ points to the dripplon (black phase), and zero otherwise.[17] The two-point correlation function:

$$R_z(r) = \langle Z(\boldsymbol{x})Z(\boldsymbol{x}+\boldsymbol{r}) - \phi^2\rangle/(\phi - \phi^2), \tag{4}$$

where $r = |\boldsymbol{r}|$ with $\boldsymbol{r}$ being a lag vector, $\langle\cdot\rangle$ denotes averaging and $\phi = \langle Z(\boldsymbol{x})\rangle$ is the fraction of dripplon area. The correlation function depends only on the modulus r of $\boldsymbol{r}$ supposing that the decomposition process is isotropic. Using the correlation function R_z, the scattering intensity $I(q)$ is then computed via Fourier transformation as:

$$I(q) = I_0 \int_0^\infty r^2 R_z(r)\frac{\sin(qr)}{qr}\mathrm{d}r, \tag{5}$$

where the constant I_0 is a normalizing factor.

We show in Supp. Fig. 3**c** the correlation function $R_z(r)$ obtained from the binary images for the three systems: $S = 22.1 \times 23.0\,\mathrm{nm}^2$, $19.7 \times 20.4\,\mathrm{nm}^2$, and $17.2 \times 17.9\,\mathrm{nm}^2$ (Supp. Fig. 3**b**). We used five images for each size in averaging in Supp. Eq. (4) to have better statistics. The obtained correlation functions are almost identical for three sizes, meaning that the property of decomposition process is insensitive to the limited box size in simulations in the range we have examined. Looking into the details, however, the range of r (limited to $< 0.3 \times L_x$ where L_x is the length of the box in x) for the small size system is not sufficiently large to capture the decay ($r \leq 5.1\,\mathrm{nm}$ for $S = 17.2 \times 17.9\,\mathrm{nm}^2$), whereas for the largest system seems sufficient. This means that, for the smaller system, the characteristic length of the pattern of the image is not sufficiently small compared with the box size. This slight difference affects the resulting scattering intensity functions as shown in Supp. Fig. 3**d**. The wavenumber at the peak is $q_m = 1.10\,\mathrm{nm}^{-1}$ for $S = 22.1 \times 23.0\,\mathrm{nm}^2$, $q_m = 1.11\,\mathrm{nm}^{-1}$ for $19.7 \times 20.4\,\mathrm{nm}^2$, and $q_m = 1.16\,\mathrm{nm}^{-1}$ for $17.2 \times 17.9\,\mathrm{nm}^2$.

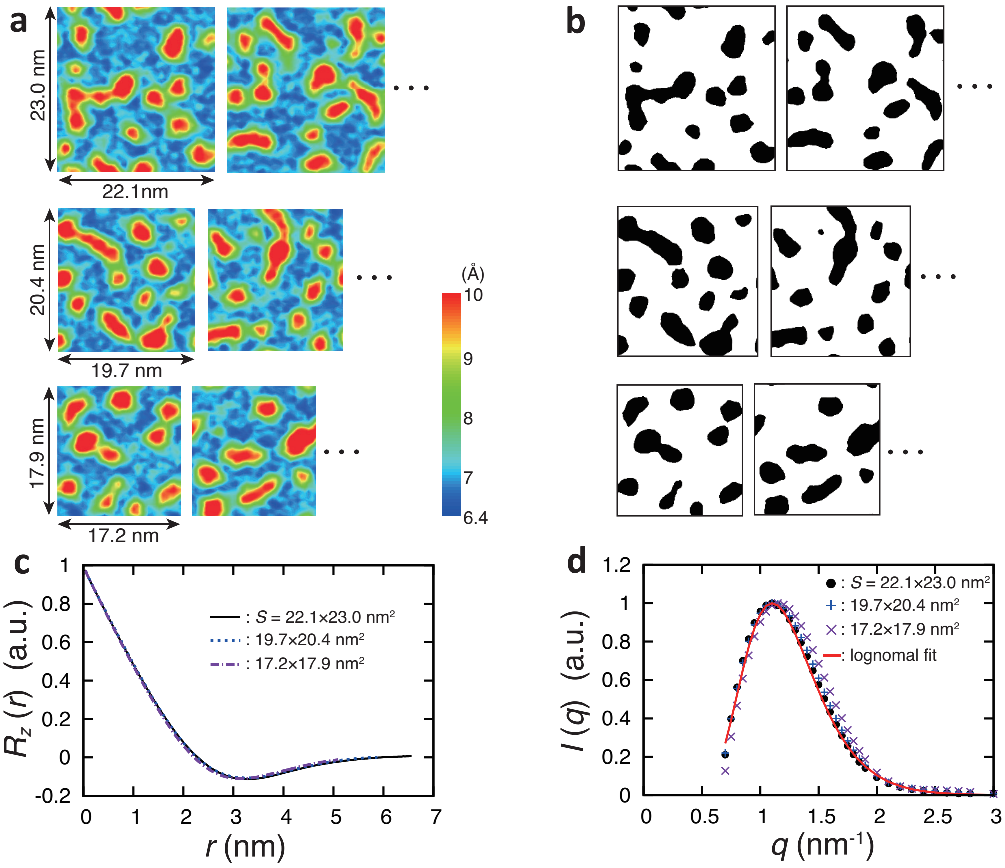

Supplementary Figure 3: **a**: Distribution of the gap between upper and lower flexible graphene sheets at $t = 5\,\mathrm{ps}$, for three different system sizes. Two images obtained with different initial configurations are shown for each size. The physical situation is the same as that in Fig. 3**a**. **b**: Binary images corresponding to panel **a**. **c**: Two-point correlation function as a function of distance r, obtained form the binary images. **d**: Scattering intensity (see Supp. Eq. (5)) as a function of wavenumber q.

Although the quantitative difference is seen for the smallest size, the value of wavenumber is well converged for the larger systems, and the effect of the box size is negligible.

Supplementary Note 3: Stabilization against Ostwald ripening

We identified the coarsening of the dripplons in the long-time regime as the Ostwald ripening. Here we discuss the stabilization using solutes trapped in the dripplon. We follow the discussion in Supp. Ref. 18. The Helmholtz free energy of a dripplon, a two-dimensional droplet accompanied by bending graphene sheets, is modeled by

$$F_d = F_b + \gamma 2\pi R, \tag{6}$$

where F_b is the free energy of water in the dripplon, while γ and R are respectively the line tension along the perimeter and the radius of the dripplon. Differentiating this expression with respect to the number of molecules results in a chemical potential given by

$$\mu_d = \mu_b + \frac{\gamma}{\rho_w R}, \tag{7}$$

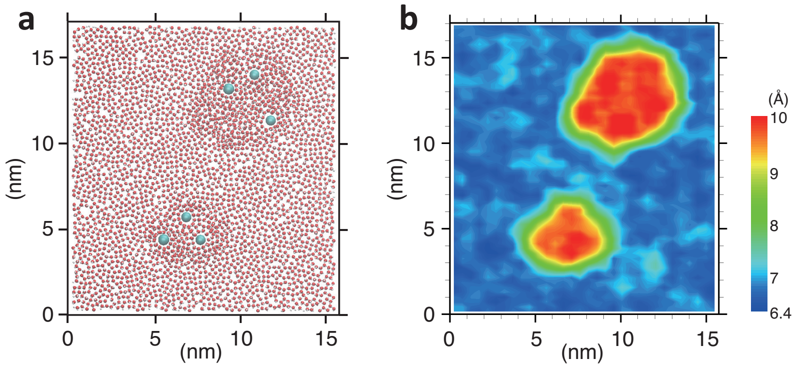

Supplementary Figure 4: **a**: Snapshot in the course of coarsening of initially two dripplons with the trapped solute particles (indicated by the blue circles.) **b**: the corresponding gap distribution. This snapshot is at $t = 0.86\,\mathrm{ns}$ of Video 3.

where ρ_w is the number density of the water molecules in the dripplon ($\rho_w \approx 20\,\mathrm{nm}^{-2}$). Now, solute particles which are only miscible in the droplet phase are considered to be trapped in the dripplon. Assuming that the trapped species is treated as dilute for simplicity, an additional term corresponding to the osmotic pressure of the trapped phase contributes to the chemical potential described above. This is expressed as

$$\Delta\mu = \frac{1}{\rho_w}\left[\frac{\gamma}{R} - \frac{N_s k_B T}{\pi R^2}\right], \tag{8}$$

where $\Delta\mu = \mu_d - \mu_b$, k_B is the Boltzmann constant, T is the temperature, and N_s is the number of solute particles trapped in the dripplon. This means that the osmotic pressure of the trapped particles given by the second term directly competes with the Laplace-like pressure described by the first term. Therefore, the equilibrium radius of the dripplon R_s with N_s solute particles is:

$$R_s = N_s k_B T/(\pi\gamma). \tag{9}$$

Now the radius of the dripplon can also be related to the number of participating water molecules $N_w \sim \rho_w \pi R_s^2$. Introducing the molar fraction of solute $x_s = N_s/(N_s + N_w)$, we can therefore rewrite Supp. Eq. (9) as:

$$R_s = \frac{\gamma}{\rho_w k_B T}\frac{1 - x_s}{x_s} = \frac{\gamma}{\rho_s k_B T}, \tag{10}$$

with ρ_s the solute number density.

For typical values $\rho_w \approx 20\,\mathrm{nm}^{-2}$, $\gamma = 3 \times 10^{-10}\,\mathrm{J/m}$ and $T = 300\,\mathrm{K}$, we obtain for a molar fraction $x_s = 10\%$ an equilibrium dripplon radius of $R_s \sim 40\,\mathrm{nm}$, as quoted in the main text. Therefore, a fully stabilized dripplon cannot be realized with a system size achiveable in molecular dynamics simulations ($S \sim 20 \times 20\,\mathrm{nm}^2$). We examined the effect of the solute particles, which represent large molecules insoluble in the water mono-layer, modeled by the electrically neutral Lennard-Jones (LJ) particles (the values of the LJ parameters are $\epsilon^{\mathrm{LJ}} = 0.42\,\mathrm{kJ/mol}$, $\sigma^{\mathrm{LJ}} = 7.0\,\text{Å}$). The coarsening process shown in Fig. 3**c** is displayed in Video 3, and a snapshot at $t = 0.86\,\mathrm{ns}$ is given in Supp. Fig. 4.

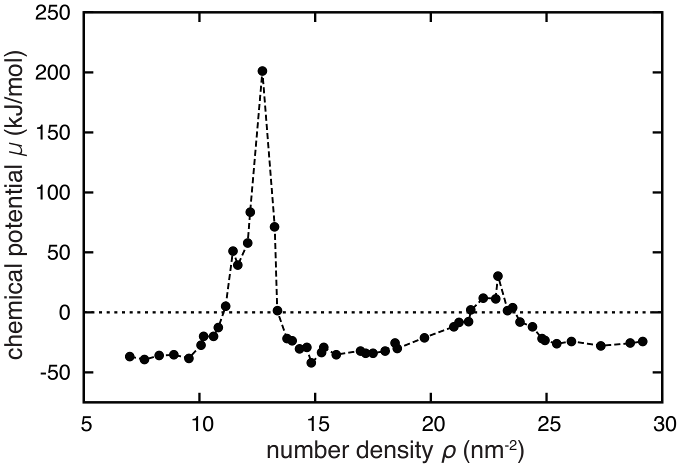

Supplementary Figure 5: Chemical potential as a function of number density ρ, computed using the test particle insertion method. The free energy shown in Fig. 4**b** is obtained by numerically integrating these data.

Supplementary Note 4: Test particle insertion method

The thermodynamic model of the dripplon described in the main text relies on the local free energy G of the water molecules confined between two graphene sheets, as a function of the number density ρ. In the present study, we numerically evaluated the chemical potential $\mu(\rho)$ using the test particle insertion method and obtained the numerical values of the free energy by integrating $\mu(\rho)$. In this section, we detail the implementation of this method (often referred to as the Widom method[19, 20]).

To access the expression for the chemical potential, we begin with the expression in the statistical mechanics for the Gibbs free energy of the system of N particles, at pressure P and temperature T:

$$G(N, P, T) = -k_B T \ln \left[\int \mathrm{d}V \frac{V^N \exp(-\beta PV)}{\lambda_d^{3N} N!} \int \mathrm{d}\boldsymbol{s}^N \exp\left(-\beta U(\boldsymbol{s}^N; V)\right) \right], \tag{11}$$

where k_B is the Boltzmann constant, $\beta = 1/k_B T$, $\lambda_d = \sqrt{h/(2\pi m_w k_B T)}$ is the thermal de Broglie wavelength with m_w the mass of a particle and h Planck's constant, and U is the potential energy. The integrals run over the possible volumes V and the configurations of the N particles (in reduced coordinates $\boldsymbol{s}^N$). We then find $\mu \, (= (\partial G/\partial N)_{PT}) = G(N+1, P, T) - G(N, P, T)$ as

$$\mu = -k_B T \ln \left\langle \frac{V}{\lambda_d^3 (N+1)} \int \mathrm{d}\boldsymbol{s}^{N+1} \exp(-\beta \Delta U) \right\rangle, \tag{12}$$

$$= \mu_{\mathrm{id}} + \mu_{\mathrm{ex}}, \tag{13}$$

where μ_{id} and μ_{ex} are the ideal and excess parts of the chemical potential, respectively, defined as

$$\mu_{\mathrm{id}} = -k_B T \ln(k_B T / P \lambda_d^3), \tag{14}$$

$$\mu_{\mathrm{ex}} = -k_B T \ln \left\langle \frac{PV}{k_B T (N+1)} \int \mathrm{d}\boldsymbol{s}^{N+1} \exp(-\beta \Delta U) \right\rangle, \tag{15}$$

and $\Delta U = U(\boldsymbol{s}^{N+1}) - U(\boldsymbol{s}^N)$ is the potential energy difference.

The practical procedure to evaluate the excess chemical potential μ_{ex} using molecular dynamics

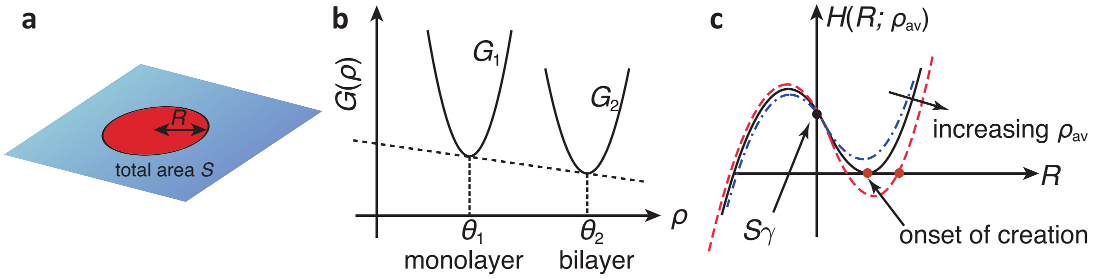

Supplementary Figure 6: **a**: Schematic of the thermodynamic model of a dripplon. **b**: Illustration of the approximated free energy as functions of the number density. **c**: Illustration of the cubic function $H(R;\rho_{\rm av})$ around the threshold ρ_c. The solid line indicates the case at the onset of the dripplon creation $\rho_{\rm av} = \rho_c$.

simulation is as follows. We perform an MD simulation for N water molecules between two rigid graphene sheets at pressure P and temperature T, as described in Supplementary Note 1. Here, we put an extra water molecule in the system, which does not interact with the other water molecules, but does with the graphene sheets such that it stays in between. During the simulation, we simply compute $U(\boldsymbol{s}^N)$, which is exactly the same as that obtained from the normal MD simulation with N molecules. Using the same trajectory, we also compute the potential energy of the system including the extra molecule, this time turning on the interaction potential between the extra molecule and the rest, which corresponds to $U(\boldsymbol{s}^{N+1})$. We then take the average in Supp. Eq. (15), over the time series of $U(\boldsymbol{s}^{N+1}) - U(\boldsymbol{s}^N)$ and V.

In obtaining Fig. 4**b**, we use the systems of small lateral sizes to prevent phase separation observed in Fig. 2**a**. Since the number of molecules is small for a small size system, the values of ρ are limited to a discrete set. Here we use two systems with $S = 1.2 \times 1.3\,\mathrm{nm}^2$ and $1.5 \times 1.3\,\mathrm{nm}^2$ to reach a sufficient resolution of ρ, as shown in Supp. Fig. 5. The free energy is then computed as $G(\rho) = \int_{\rho_0}^{\rho} \mu(\rho')\mathrm{d}\rho'$, which is shown in Fig. 4**b** in the paper.

Supplementary Note 5: Thermodynamic model of a dripplon

In this section, we discuss the solution procedure for the thermodynamic model presented in the main text. We repeat here the expression of the free energy model for a single dripplon in the system (see Supp. Fig. 6**a**):

$$G_{\rm total} = (S - \pi R^2)G_1 + \pi R^2 G_2 + 2\pi R\gamma, \tag{16}$$

where S is the total area, $G_1(\rho_1)$ and $G_2(\rho_2)$ are the free energies per area of the mono- and bi-layer phases, respectively, and γ is the line tension, *i.e.* the energy per unit length of the perimeter of the dripplon. In order to solve the problem analytically, we approximate G_1 and G_2 by quadratic functions of the density: $G_i(\rho_i) = C_i(\rho_i - \theta_i)^2 + b\rho_i + d$, as illustrated in Supp. Fig. 6**b**. This approximation is reasonable given the numerical free energy as a function of ρ as in Fig. 4**b**, and greatly simplifies the analysis of the model. The parameters C_i and θ_i are identified as $C_1 = 33.4\,\mathrm{kJ\ nm^2/mol}$, $C_2 = 14.6\,\mathrm{kJ\ nm^2/mol}$, $\theta_1 = 10.9\,\mathrm{nm}^{-2}$, and $\theta_2 = 21.5\,\mathrm{nm}^{-2}$. ($b$ and d disappear in the analysis).

Since the total number of water molecules is fixed at $S\rho_{\rm av}$ in our system, we have an additional

constraint: $(S-\pi R^2)\rho_1+\pi R^2\rho_2=\rho_{\rm av}S$. The radius of the dripplon R is then obtained by means of the Lagrange multiplier method, minimizing the total free energy $G_{\rm total}(\rho_1,\rho_2,R)$. Let us define the following Lagrange function

$$\mathcal{L}(\rho_1,\rho_2,R)=(S-\pi R^2)G_1+\pi R^2 G_2+2\pi R\gamma+\lambda\left[(S-\pi R^2)\rho_1+\pi R^2\rho_2-\rho_{\rm av}S\right]. \tag{17}$$

The conditions satisfied at the local minimum (maximum) of $G_{\rm total}$, *i.e.*, $\partial\mathcal{L}/\partial\rho_i=0$, $\partial\mathcal{L}/\partial R=0$, and $\partial\mathcal{L}/\partial\lambda=0$, yield the following equations:

$$\Delta C\tilde{\lambda}^2-4\tilde{\lambda}\Delta\theta-4\gamma/R=0, \tag{18}$$

$$\pi\Delta\theta R^2-\pi\Delta C\tilde{\lambda}R^2/2-S\tilde{\lambda}/(2C_1)+(\theta_1-\rho_{\rm av})S=0, \tag{19}$$

$$\rho_1=\theta_1-\tilde{\lambda}/(2C_1), \tag{20}$$

$$\rho_2=\theta_2-\tilde{\lambda}/(2C_2), \tag{21}$$

where $\Delta C=1/C_2-1/C_1$, $\Delta\theta=\theta_2-\theta_1$, and $\tilde{\lambda}=\lambda-b$. For a given value of $\rho_{\rm av}$, Supp. Eqs. (18) and (19) are solved with respect to R by eliminating $\tilde{\lambda}$. The result of this solution, referred to as the full model here, is plotted in Fig. 4**a** in the paper. Note that this model reduces to the lever rule if we assume no line tension $\gamma=0$:

$$\pi\Delta\theta R^2+(\theta_1-\rho_{\rm av})S=0, \tag{22}$$

with $\tilde{\lambda}=0$, $\rho_1=\theta_1$, and $\rho_2=\theta_2$.

At the onset of the creation of the dripplon, the shape of G_1 is expected to play more important role than that of G_2. Hence we attempt to further simplify the model, assuming $C_2=C_1$, or $\Delta C=0$. Then Supp. Eqs. (18) and (19) reduce to

$$H(R;\rho_{\rm av})=2\pi C_1\Delta\theta^2R^3+2C_1\Delta\theta(\theta_1-\rho_{\rm av})SR+S\gamma=0. \tag{23}$$

The behavior of this cubic function H is illustrated in Supp. Fig. 6**c**; Supp. Eq. (23) has roots only for $\rho_{\rm av}>\rho_c$, where the threshold ρ_c is given by equating the discriminant to zero:

$$\rho_c=\theta_1+\frac{3}{2}\left(\frac{\pi\gamma^2}{2\Delta\theta C_1^2 S}\right)^{1/3}. \tag{24}$$

With the choice of $\gamma=6.1\times10^{-10}$ J/m as quoted in the main text, the threshold for Fig. 4**a** is $\rho_c=11.5\,\mathrm{nm}^{-2}$, which perfectly matches the MD data. We show additional data for different total areas S in Supp. Fig. 7: $S=11.8\times12.8\,\mathrm{nm}^2$, $15.7\times17.0\,\mathrm{nm}^2$, and $19.7\times21.3\,\mathrm{nm}^2$, here along with the simplified model given by Supp. Eq. (23). We see excellent agreement between the MD data and the thermodynamic models; the difference between the full model and simplified one is negligible.

Our thermodynamic model presented given in Supp. Eq. (16) shows no upper bound for the size of the dripplons, as the radius of the dripplons increases as $R\sim\sqrt{\rho_{\rm av}}$ for large $\rho_{\rm av}$ (cf. Supp. Eq. (22)). In practical situations, however, the size of the numerical box L should remain larger than the dripplon diameter to avoid finite size effect. As shown in Supp. Fig. 7d, in such a situation (here for $S=15.7\times17.0\,\mathrm{nm}^2$ and $\rho_{\rm av}=14.3\,\mathrm{nm}^{-2}$), the dripplon remains but take

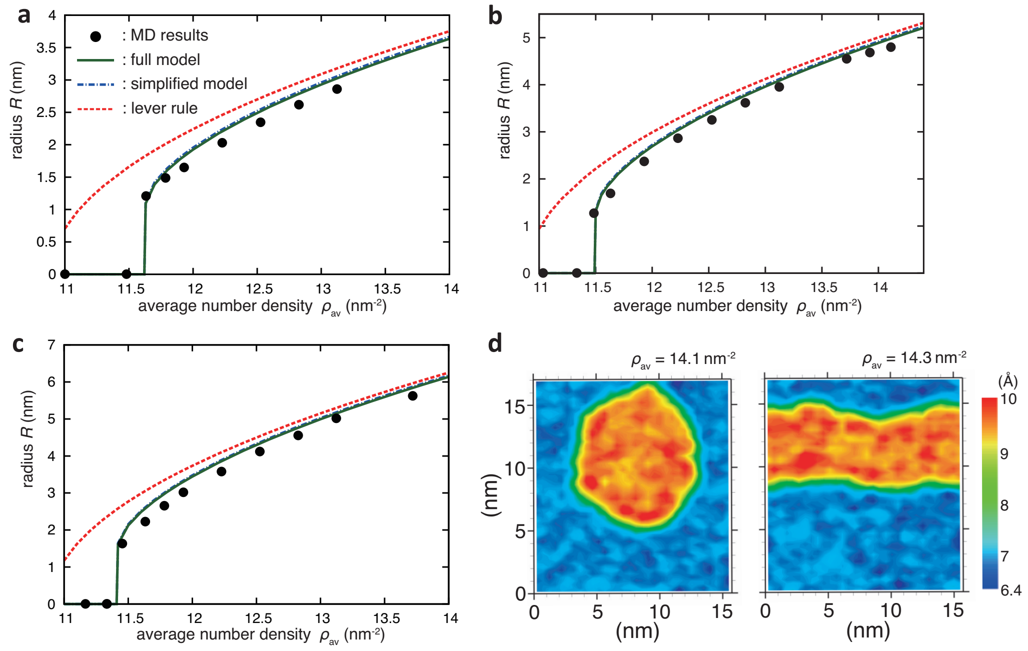

Supplementary Figure 7: Radius of the dripplon R versus average number density $\rho_{\rm av}$, for the system size of $S = 11.8 \times 12.8\,{\rm nm}^2$ (**a**) $15.7 \times 17.0\,{\rm nm}^2$ (**b**) and $19.7 \times 21.3\,{\rm nm}^2$ (**c**). The solid line indicates the prediction of the full model given in Supp. Eqs. (18) and (19), and the dash-doted line indicates the simplified model given in Supp. Eq. (23). The prediction of the lever rule is shown by the dashed line. **d**: Gap distribution between upper and lower flexible graphene sheets at $\rho_{\rm av} = 14.1$ and $14.3\,{\rm nm}^{-2}$.

a stripe geometry instead of a circular geometry. This is expected in order to minimize the corresponding line energy in a finite size box.

Supplementary Note 6: Extended data for dripplon dynamics

We show here extended data supplemental to the discussion on the dripplon dynamics. The diffusion coefficient discussed in Fig. 5 is computed via the two dimensional mean square displacement (MSD) of the dripplon. Specifically, we track the center-of-mass of the water molecules inside the dripplon, and compute $\langle \Delta r^2 \rangle$, where $\Delta r = (\Delta x^2 + \Delta y^2)^{1/2}$ is the in-plane displacement. For each dripplon, we carried out simulations longer than 3×10^6 time steps (3 ns), for the system of $S = 15.7 \times 17.0\,{\rm nm}^2$, with 10240 carbon atoms in each flexible graphene sheet and more than 30000 water molecules in total. In addition to the MSD shown in Fig. 5**c**, we plot in Supp. Fig. 8**a** the MSD for five dripplons considered in the inset of Fig. 5**c**. In the long-time regime (hundreds of ps), the MSD scales as $\langle \Delta r^2 \rangle \sim \Gamma t^\alpha$, with $\alpha \approx 1.3$ for all the dripplons. Therefore, the dynamics of the dripplon exhibits a slight superdiffusive behavior[21] as discussed in the main text, rather than pure Brownian motion. Hence the apparent diffusion coefficients defined as $D_{\rm app} = \Gamma t_0^{\alpha-1}/4$ with $t_0 = 0.2\,{\rm ps}$[22] are plotted in the inset of Fig. 5**c**.

On the other hand, the MSD of each confined water molecule inside and outside the dripplon exhibits a purely Brownian behavior, as quoted in the main text. In Supp. Fig. 8**b** we plot the (two dimensional) MSD of water molecules. The long-time behavior shows clear linearity. Thus the self-diffusion coefficients are obtained from the slope of the MSD as $\langle \Delta r^2 \rangle \sim 4Dt$. The resulting values $D = 2.4 \times 10^{-5}\,{\rm cm}^2/{\rm s}$ inside the dripplon (bi-layer), and $4.2 \times 10^{-5}\,{\rm cm}^2/{\rm s}$ outside the

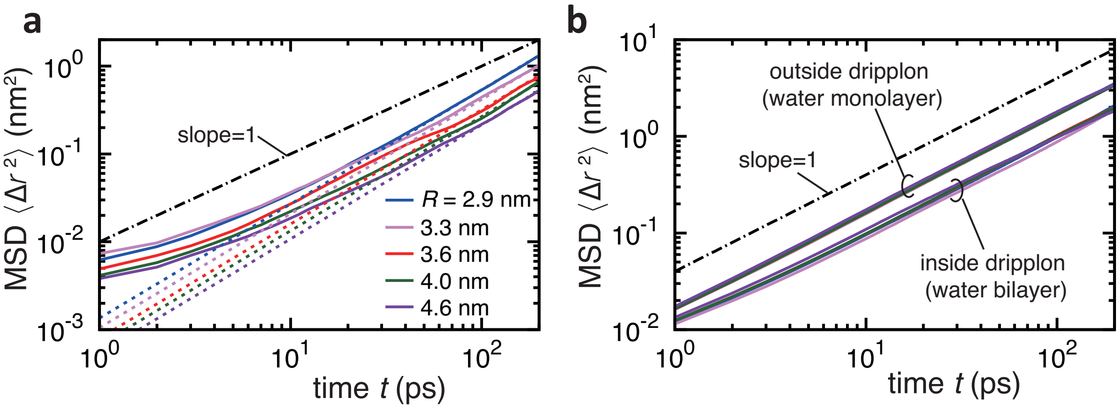

Supplementary Figure 8: **a**: Log-log plot of the MSD of the dripplon as a function of time. The dotted lines are fits to $\langle \Delta r^2 \rangle \sim \Gamma t^\alpha$, with $\alpha = 1.3$ in the long-time regime. **b**: Same plot for individual water molecules inside and outside the dripplon. A line with slope equal to unity is shown in each figure by the dash-dotted line.

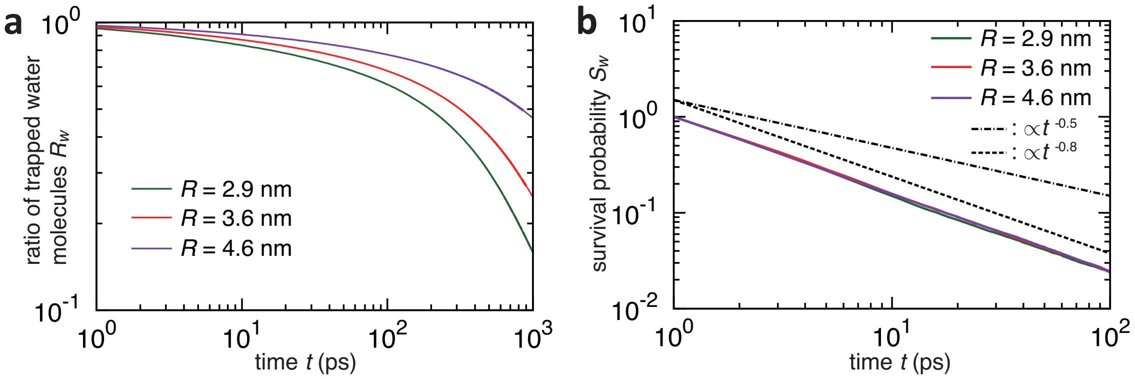

Supplementary Figure 9: **a**: Ratio of water molecules trapped in the dripplon, defined as $R_w(t) = N_w(t)/N_w(0)$, where $N_w(t)$ is the number of water molecules in the dripplon at t, which have been staying since $t = 0$ without crossing the interface. **b**: Survival probability within the dripplon. $S_w(t)$ indicates the probability that a water molecule participates in the dripplon for longer than t (larger than 1 ps).

dripplon (mono-layer) are in good agreement with the reported values for the water mono-layer and bi-layer.[23]

We next show the data proving the exchanges of water molecules participating the dripplon. The plot in Supp. Fig. 9**a** shows the quantitative counterpart demonstrated in Figs. 5**a** and **b** and Video 4, *i.e.* the ratio defined as $R_w(t) = N_w(t)/N_w(0)$, where $N_w(t)$ is the number of water molecules found in the dripplon at t, which remained in the dripplon since $t = 0$; the molecules that re-entered in the dripplon are not counted. For the dripplon of $R = 2.9\,\text{nm}$, 84 % of water molecules go out within 1 ns.

In Supp. Fig. 9**b** we show the survival probability function $S_w(t)$ for the water molecules belonging to the dripplon. This is defined such that $S_w(t)$ is the probability that a water molecule stays in the dripplon longer than t. To numerically evaluate this function, we compute the probability $P_w(t)\mathrm{d}t$ that a water molecule belongs to the dripplon for a time period t, by measuring

the period for which each water molecule stays inside after entering the dripplon. Here, only the molecules that stays at least for 1 ps are counted. The survival probability is then computed as $S_w(t) = 1 - \int_0^t P_w(s)\mathrm{d}s$. The plot in Supp. Fig. 9**b** displays a monotonic decay down to less than 3 % at 100 ps. The fact that the survival probability only slightly depends on the radius of the dripplon indicates the very active exchanges of water molecules near the interface. We remark that, if the motion of dripplon was pure Brownian diffusion, this survival probability should decay as $t^{-1/2}$. This is shown via an argument parallel with the following paragraph on S_c, using the fact that the water molecules themselves exhibit pure Brownian motion (Supp. Fig. 8**b**). Therefore the observed faster decay ($\sim t^{-0.8}$) of $S_w(t)$ (Supp. Fig. 9**b**) is an indication of departure from normal diffusion, on top of the decay of S_c discussed in the main text. Here (and in Fig. 5**d** of the main text) the decay rate $t^{-1/2}$ was checked using a simple simulation of a single disk undergoing pure Brownian motion on the 2D plane; grid points are fixed on the 2D plane, and the survival probability was computed by checking whether these grid points are inside or outside the disk.

Supplementary References

[1] J. L. F. Abascal and C. Vega, "A general purpose model for the condensed phases of water: TIP4P/2005," J. Chem. Phys. **123**, 234505 (2005).

[2] S. L. Mayo, B. D. Olafson, and W. A. Goddard III, "Dreiding: A generic force field for molecular simulations," J. Phys. Chem. **94**, 8897 (1990).

[3] M. P. Allen and D. J. Tildesley, *Computer Simulation of Liquids*, Oxford Univ. Press, Oxford, 1989.

[4] See `http://lammps.sandia.gov` for the code.

[5] J.-P. Ryckaert, G. Ciccotti, and H. J. C. Berendsen, "Numerical integration of the cartesian equations of motion of a system with constraints: molecular dynamics of n-alkanes," J. Comput. Phys. **23**, 327 (1977).

[6] I.-C. Yeh and M. L. Berkowitz, "Ewald summation for systems with slab geometry," J. Chem. Phys. **111**, 3155 (1999).

[7] M. Ma, G. Tocci, A. Michaelides, and G. Aeppli, "Fast diffusion of water nanodroplets on graphene," Nature Mater. **15**, 66 (2016).

[8] Y. Liu, Z. Xu, and Q. Zheng, "The interlayer shear effect on graphene multilayer resonators," J. Mech. Phys. Solid. **59**, 1613 (2011).

[9] R. Nicklow, N. Wakabayashi, and H. G. Smith, "Lattice dynamics of pyrolytic graphite," Phys. Rev. B **5**, 4951 (1972).

[10] W. D. Cornell, P. Cieplak, C. I. Bayly, I. R. Gould, K. M. Merz, D. M. Ferguson, D. C. Spellmeyer, T. Fox, J. W. Caldwell, and P. A. Kollman, "A second generation force field for the simulation of proteins, nucleic acids, and organic molecules," J. Am. Chem. Soc. **117**, 5179 (1995).

[11] H. J. C. Berendsen, J. R. Grigera, and T. P. Straatsma, "The missing term in effective pair potentials," J. Phys. Chem. **91**, 6269 (1987).

[12] T. Werder, J. H. Walther, R. L. Jaffe, T. Halicioglu, and P. Koumoutsakos, "On the water-carbon interaction for use in molecular dynamics simulations of graphite and carbon nanotubes," J. Phys. Chem. B **107**, 1345 (2003).

[13] M. A. González and J. L. F. Abascal, "A flexible model for water based on TIP4P/2005," J. Chem. Phys. **135**, 224516 (2011).

[14] A. Vrij, "Possible mechanism for the spontaneous rupture of thin, free liquid films," Discuss. Faraday Soc. **42**, 23 (1966).

[15] J. E. Coons, P. J. Halley, S. A. McGlashan, and T. Tran-Cong, "A review of drainage and spontaneous rupture in free standing thin films with tangentially immobile interfaces," Adv. Colloid Interf. Sci. **105**, 3 (2003).

[16] A. P. Radlinski, M. A. Ioannidis, A. L. Hinde, M. Hainbuchner, M. Baron, H. Rauch, and S. R. Kline, "Angstrom-to-millimeter characterization of sedimentary rock microstructure," J. Colloid. Interf. Sci. **274**, 607 (2004).

[17] P. Adler, C. Jacquin, and J. Quiblier, "Flow in simulated porous media," Int. J. Multiphase Flow **16**, 691 (1990).

[18] A. J. Webster and M. E. Cates, "Stabilization of emulsions by trapped species," Langmuir **14**, 2068 (1998).

[19] D. Frenkel and B. Smit, *Understanding molecular simulation: from algorithms to applications*, Academic Press, 2002.

[20] B. Widom, "Some topics in the theory of fluids," J. Chem. Phys. **39**, 2808 (1963).

[21] R. Metzler and J. Klafter, "The random walk's guide to anomalous diffusion: A fractional dynamics approach," Phys. Rep. **339**, 1 (2000).

[22] T. J. Feder, I. Brust-Mascher, J. P. Slattery, B. Baird, and W. W. Webb, "Constrained diffusion or immobile fraction on cell surfaces: A new interpretation.," Biophys. J. **70**, 2767 (1996).

[23] R. Zangi, "Water confined to a slab geometry: A review of recent computer simulation studies," J. Phys. Cond. Mat. **16**, S5371 (2004).
