# Supplementary material for: Dripplons as localized and superfast ripples of water confined between graphene sheets
Source: Nat Commun. 2018 Apr 16;9:1496. doi: 10.1038/s41467-018-03829-1 (PMC5902618; doi:10.1038/s41467-018-03829-1)
Supplement: Supplementary file 3 — Description of Additional Supplementary [file 41467_2018_3829_MOESM3_ESM.pdf]

## Description of Additional Supplementary Files

## Dripplons as localized and superfast ripples of water confined between graphene sheets

Yoshida *et al.*

**Supplementary Movie 1:** Overview of the motion of flexible graphene sheets, starting from the homogeneous state at $h = 0.85\,\mathrm{nm}$. The gap distribution is also shown. The system size is $S = 19.7 \times 20.4\,\mathrm{nm}^2$ and the average density is $\rho_{\mathrm{av}} = 12.1\,\mathrm{nm}^2$.

**Supplementary Movie 2:** Time evolution shown in Fig. 3**a**, *i.e.* the evolution of the top views of the water molecules between flexible graphene sheets along with the distribution of gap between upper and lower flexible graphene sheets. The situation is the same as Video 1, except that the system size is $S = 22.1 \times 23.0\,\mathrm{nm}^2$.

**Supplementary Movie 3:** Time evolution shown in Fig. 3**c**, *i.e.* the coarsening process of initially two dripplons in the graphene sheets of $S = 15.7 \times 17.0\,\mathrm{nm}^2$. The average density is $\rho_{\mathrm{av}} = 12.5\,\mathrm{nm}^2$. For comparison, the case in which solute particles are trapped in the dripplons, discussed in Supplementary Note 3, is also shown.

**Supplementary Movie 4:** Motion of water molecules shown in Figs. 5**a** and **b**, *i.e.* the top view of the equilibrium dripplon at $\rho_{\mathrm{av}} = 12.3\,\mathrm{nm}^2$ in the sheets of $S = 15.7 \times 17.0\,\mathrm{nm}^2$, with the water molecules belonging to the dripplon at $t = 0$ colored in red. The corresponding gap distribution is shown as a guide.
